# Hybrid Coatings Enriched with Tetraethoxysilane for Corrosion Mitigation of Hot-Dip Galvanized Steel in Chloride Contaminated Simulated Concrete Pore Solutions

**DOI:** 10.3390/ma10030306

**Published:** 2017-03-17

**Authors:** Rita B. Figueira, Emanuela Callone, Carlos J. R. Silva, Elsa V. Pereira, Sandra Dirè

**Affiliations:** 1 LNEC, National Laboratory for Civil Engineering, Av. do Brasil 101, Lisboa 1700-066, Portugal; epereira@lnec.pt; 2 “Klaus Müller” NMR Laboratory, Department of Industrial Engineering, University of Trento, Via Sommarive, 9, Trento 38123, Italy; emanuela.callone@unitn.it (E.C.); sandra.dire@unitn.it (S.D.); 3 Centro de Química, University of Minho, Campus de Gualtar, Braga 4710-057, Portugal; csilva@quimica.uminho.pt

**Keywords:** sol-gel, hybrids, coatings, corrosion mitigation, alkaline environment, chlorides

## Abstract

Hybrid sol-gel coatings, named U(X):TEOS, based on ureasilicate matrices (U(X)) enriched with tetraethoxysilane (TEOS), were synthesized. The influence of TEOS addition was studied on both the structure of the hybrid sol-gel films as well as on the electrochemical properties. The effect of TEOS on the structure of the hybrid sol-gel films was investigated by solid state Nuclear Magnetic Resonance. The dielectric properties of the different materials were investigated by electrochemical impedance spectroscopy. The corrosion behavior of the hybrid coatings on HDGS was studied in chloride-contaminated simulated concrete pore solutions (SCPS) by polarization resistance measurements. The roughness of the HDGS coated with hybrids was also characterized by atomic force microscopy. The structural characterization of the hybrid materials proved the effective reaction between Jeffamine^®^ and 3-isocyanate propyltriethoxysilane (ICPTES) and indicated that the addition of TEOS does not seem to affect the organic structure or to increase the degree of condensation of the hybrid materials. Despite the apparent lack of influence on the hybrids architecture, the polarization resistance measurements confirmed that TEOS addition improves the corrosion resistance of the hybrid coatings (U(X):TEOS) in chloride-contaminated SCPS when compared to samples prepared without any TEOS (U(X)). This behavior could be related to the decrease in roughness of the hybrid coatings (due TEOS addition) and to the different metal coating interaction resulting from the increase of the inorganic component in the hybrid matrix.

## 1. Introduction

Corrosion of reinforcement is one of the major causes of damage of reinforced concrete structures (RCS) worldwide [1,2,3,4,5,6]. The durability requirements, particularly in harsh environments such as marine and industrial, are not always achieved [5,7,8,9,10]. Generally, the major causes for corrosion of reinforcement in concrete are carbonation of concrete due to concrete acidification after reaction with atmospheric carbon dioxide, and/or the presence of chloride ions. In marine environments, chloride ions can penetrate the porous structure of concrete and reach the steel [6]. In other cases, a recurrent malpractice is the use of chloride-contaminated coarse aggregates and water constituents leading to the presence of chloride ions in the concrete since the beginning [11,12].

The reliability and the durability of RCS are crucial for the society and its economy due to the high costs of structure conservation and maintenance. Therefore, in order to increase their service life in aggressive environments, several methods have been proposed [7,13,14,15]. The use of hot-dip galvanized steel (HDGS) has been widely recognized as an effective measure to increase the service life of RCS [15,16,17]. Nevertheless, when the HDGS is in contact with concrete pore solutions, whose pH is typically above 12.5, zinc corrosion occurs [16,18,19,20,21,22,23,24]. This leads to zinc layer consumption until either, a passivation layer is formed, or until the entire zinc layer is consumed. Commonly, to mitigate this initial corrosion process, procedures such as increasing the chromate content of the concrete mixture or the use of chromate conversion layers have been widely applied. However, due to the hard restrictions imposed on the use of Cr(VI), a growing interest in developing innovative materials for chromate conversion layers replacement has led to the synthesis and assessment of several organic–inorganic hybrid sol-gel materials [25,26,27,28,29,30,31].

Ureasilicate (U(X)) coatings were proven to behave as a physical barrier in highly alkaline environments (pH > 12.5) [32,33,34,35] and to be effective in hindering the chloride ion diffusion to the substrate. Therefore, these materials mitigate the corrosion reaction rate on the metal surface of HDGS in chloride contaminated SCPS [36]. U(X) hybrids are prepared from 3-isocyanate propyltriethoxysilane (ICPTES) and di-amino functionalized polyether (hereafter referred generically as Jeffamine^®^) [27,33,34,35]. The polymeric chains of these polyetheramines are block co-polymers based on propylene-ethylene-ethylene glycols sequences. The Jeffamine^®^ molecules establish a chemical link with the inorganic network created by the condensation reactions of the organosilane [32]. Improved performance was obtained for U(X) samples prepared with lower molecular weight (MW) of Jeffamine^®^ (≈230 and 400 g·mol^−1^) [27,33,34,35] rather than with samples prepared with higher MWs of Jeffamine^®^ (≈600, 900 and 2000 g·mol^−1^) [32].

This study aimed to improve the corrosion protection properties of U(X) materials, prepared with high MWs of Jeffamine^®^ by increasing the inorganic component of the hybrid matrices both by increasing the content of ICPTES [37] and adding tetraethoxysilane (TEOS). Therefore, new U(X) compositions were obtained employing a Jeffamine:ICPTES molar ratio 1:4.16 and adding controlled amounts of TEOS into the reaction mixture. The obtained hybrid coatings were tested in alkaline environments.

TEOS was chosen because its properties and reactivity are quite well known at different pH and temperatures [25,38,39]. It is a silica precursor available with a higher purity grade, presents a relatively slow and controllable rate of reaction, and it is less toxic when compared to tetramethoxysilane (TMOS). Organic–inorganic hybrids based on TEOS can produce, at low temperatures, homogeneous films on large areas of substrates, with improved transparency [25]. Moreover, organic–inorganic hybrids based on TEOS are low cost. Indeed, this precursor, for similar purity grades, is about four times cheaper than organosilanes commonly employed such as 3-glycidoxypropyltrimethoxy-silane (GPTMS) and half of the price of methyltriethoxysilane (MTES) [25].

The structural features of the U(X) sol-gel films and the effect of TEOS addition were investigated by solid state Nuclear Magnetic Resonance. The effect of increasing the inorganic components on the dielectric properties of the hybrid films was studied by electrochemical impedance spectroscopy to determine the respective conductivity and dielectric constant. The electrical properties are strongly affected by the structural matrix of the hybrid materials. The effect of stiffening induced by the silicate chains resulting from the condensation of inorganic components is opposed by the liquid-like properties from the presence of organic chain components. Therefore, the kinetics and extension of ions movement across materials is dependent on the organic and inorganic components concentration and interactions. The corrosion resistance of the different hybrid coatings synthesized (with and without TEOS) was investigated, in chloride-contaminated simulated concrete pore solutions, by polarization resistance. The morphology of the coatings (roughness) was also assessed by atomic force microscopy.

## 2. Experimental Section

### 2.1. Materials

Jeffamine^®^ ED-600, Jeffamine^®^ ED-900 and Jeffamine^®^ ED-2000 (Fluka), 3-Isocyanate propyltriethoxysilane (ICPTES, 95%, Aldrich) and tetraethoxysilane (TEOS, 98%, Aldrich) were purchased from Sigma-Aldrich, Química, Sintra, Portugal, and used as supplied. Absolute ethanol (EtOH, absolute 98%, Riedel-de-Haën (Sigma-Aldrich Química, Sintra, Portugal) and citric acid monohydrate (Merck, Darmstadt, Germany) were likewise used as provided. Ultra-pure water (0.055–0.060 μS/cm) obtained from a Purelab Ultra System (Elga, (Lab Water-Veolia Water Solutions & Technologies, Paris, France)) was used.

### 2.2. Sol-Gel Synthesis of Hybrid Ureasilicate Coatings

**Figure 1:**
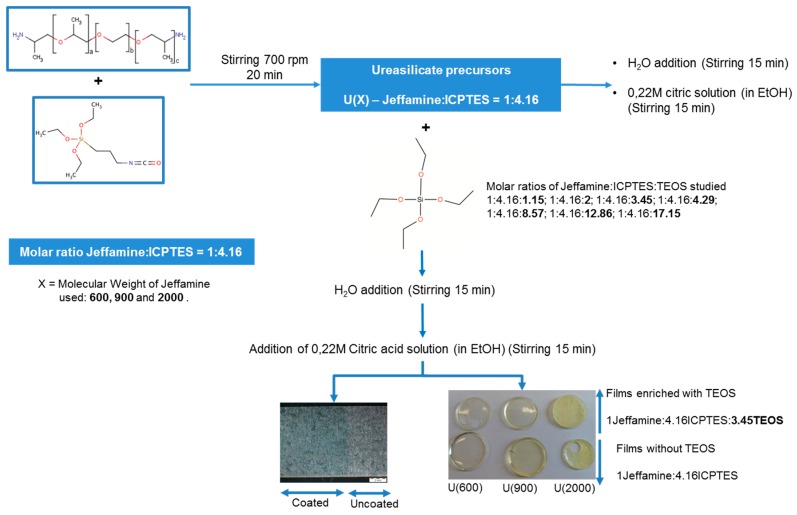
Schematic representation of the main steps involved in the production of hybrid films and coatings with and without tetraethoxysilane (TEOS).

The experimental steps involved in the synthesis of the hybrid sol-gel matrices, to produce films and coatings on HDGS samples, are shown in Figure 1.

Different materials were prepared as films or coatings on HDGS with and without TEOS (U(600), U(600):TEOS, U(900), U(900):TEOS, U(2000) and U(2000):TEOS). Three U(X) precursors were obtained by mixing a stoichiometric amount (1:4.16) of Jeffamine^®^ (ED-600, ED-900 and ED-2000) and ICPTES in a closed vessel (Table 1). Other materials with different molar ratios of Jeffamine:TEOS were prepared. In all the syntheses, the molar ratios H_2_O:Jeffamine and Jeffamine:Citric Acid were kept constant by controlling the amounts of 0.2 M Citric acid solution and water added. The synthesis was performed using a standard procedure as described in literature [27,32,36]. The samples synthesis was performed with adding water in stoichiometric amount to hydrolyze the ethoxyde groups of both ICPTES and TEOS.

For U(X) matrices that were enriched with TEOS, the appropriate quantity was added to each ureasilicate, according to the molar ratios indicated in Table 1 (U(600):TEOS, U(900):TEOS and U(2000):TEOS). In the third step, 0.22 M solution of citric acid in ethanol solution was added and stirred during 15 min. After that, distilled water was added and the final mixture was left to react for further 15 min.

### 2.3. Preparation of the Hybrid Films and Coatings on Hot-Dip Galvanized Steel

Hybrid matrices were synthesized to produce films or coatings deposited on HDGS substrate. The hybrid films were prepared according to the ratios indicated in Table 1. The coatings prepared without TEOS on HDGS were U(600), U(900) and U(2000); all with a molar ratio of Jeffamine:ICPTES equal to 1:4.16. For the samples enriched with TEOS, only the ones using a ratio of Jeffamine:TEOS equal to 1:3.45 (identified by U(600):3.45TEOS, U(900):3.45TEOS and U(2000):3.45TEOS) were prepared as coatings.

Both coatings and films samples, based on U(X) and U(X) enriched with TEOS, identified as U(X):TEOS, were produced from a single batch of precursor solution (in a sol form). For the preparation of the U(X) films, the remainder of the prepared mixture was transferred to a petri dish (polystyrene, 2 cm of diameter, supplied by Sarstedt). All the U(X) enriched with TEOS (U(X):TEOS) films (Table 1) were transferred to a *Teflon*™ mold (with an inner diameter of 3 cm) and covered with *Parafilm*^®^.

The HDGS samples were obtained from commercially available plates and cut to dimensions of 5.0 cm × 1.0 cm × 0.1 cm. The HDGS samples had an average zinc thickness of 16 μm on both sides. Before coating deposition, the HDGS metal plates were degreased with acetone and dried at room temperature. Coated HDGS samples were prepared by dipping the metallic plates of HDGS in the synthesized sol mixture using a dip coater (Nima, model DC Small). The hybrid coatings were deposited by one and three consecutive dip steps at a withdrawal speed of 10 mm·min^−1^, without residence time. Producing samples coated by either one or three consecutive dip steps, allowed the assessment of performance against corrosion of thinner and thicker coatings in chloride-contaminated SCPS. All the synthesized hybrid films and the coated HDGS samples were immediately placed in an incubator-compressor (ICP-400 Memmert, (Memmert GmbH + Co. KG, Schwabach, Germany)) and kept at 40 °C for 15 days. The films for the solid state NMR analyses, after the curing process, were removed from the petri dishes (U(X)) and from the *Teflon*™ mold (U(X):TEOS) and smashed to form a powder.

### 2.4. Structural Characterization of the Hybrid Materials by Solid State Nuclear Magnetic Resonance (NMR)

Solid state NMR analyses were carried with a Bruker 400 WB spectrometer operating at a proton frequency of 400.13 MHz. NMR spectra were acquired under the following conditions: ^13^C frequency: 100.48 MHz, π/2 pulse 3.4 μs cross polarization sequence, contact time 2000 µs, decoupling length 6.3 µs, recycle delay: 5 s, 5 k scans. ^29^Si frequency: 79.48 MHz, π/2 pulse 3.9 μs. Single pulse sequence: π/4 pulse 3.9 μs, decoupling length 6.3 μs, recycle delay 100 s, 2 k scans. Samples were packed in 4 mm zirconia rotors, which were spun at 9 kHz under air flow. Adamantane and Q_8_M_8_ were used as external secondary references. Liquid NMR data were recorded on a Bruker 400 WB spectrometer operating at a proton frequency of 400.13 MHz equipped with a 5 mm BBO probe under the following conditions: ^13^C frequency: 100.48 MHz, power gated single pulse sequence, π/6 pulse 2.7 µs, with 80 µs waltz decoupling, recycle delay: 30 s, 128 scans. Bruker TopSpin software was used for the lineshape analysis. The results were considered acceptable with confidence level of 95%.

### 2.5. Glow Discharge Optical Emission Spectroscopy (GD-OES)

The chemical composition depth profiling of the coatings applied on the HDGS substrates was performed using a glow discharge optical emission spectrometer on coated and uncoated substrates. A LECO glow discharge GD OES 850A, with a radiofrequency source and a 700 V RMS was used and the samples were analyzed under argon atmosphere.

### 2.6. Characterization of the Dielectric Properties of the Hybrid Films by Electrochemical Impedance Spectroscopy (EIS)

**Figure 2:**
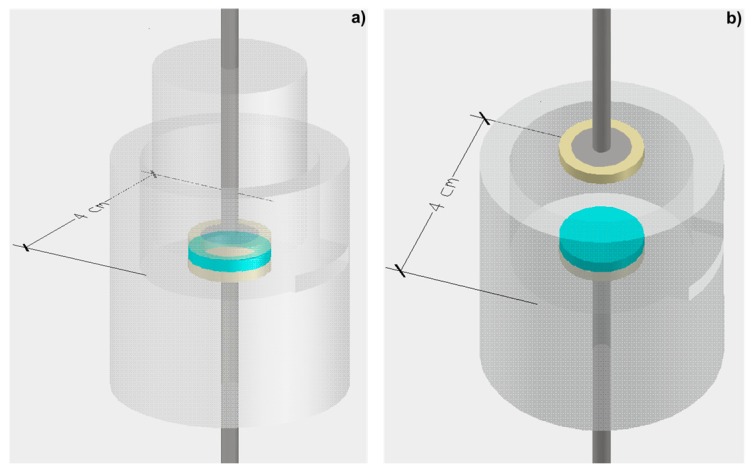
(**a**) Image of the support cell for Electrochemical Impedance Spectroscopy (EIS) measurements; and (**b**) top view of the cell. The blue disc represents the hybrid film that is placed between the two Au electrodes (adapted from [40]).

EIS measurements were carried out to characterize the electrical impedance, dielectric constant and capacitance of the prepared hybrid films. Two Au disc electrodes (10 mm diameter and 250 µm thickness) and a support cell (Figure 2) adapted from a previous model [40] were used. All measurements were performed at room temperature using an Impedance/Gain-Phase Analyzer (Model 1260A, Solartron-Schumberger) and a Potentiostat/Galvanostat (Model 1287A, Solartron-Schlumberger (AMETEK, Inc., Berwyn, PA, USA)) controlled by a PC using Zplot software (Solartron-Schlumberger, version 2.9c). Measurements were taken by applying a 10 mV (peak-to-peak, sinusoidal) electrical potential within a frequency range from 1 × 10^5^ Hz to 0.01 Hz (10 points per decade) between the two Au electrodes at open circuit potential. The frequency response data of the hybrid films studied were displayed in a Nyquist plot, using ZView software (Solartron-Schlumberger, version 2.9c) that was also used for data fitting purposes.

### 2.7. Surface Characterization of the Hybrid Coatings Deposited on HDGS

The morphology of the hybrid sol-gel coatings applied on HDGS specimens was studied by atomic force microscopy (AFM). The AFM images were taken operating in air using the dimension NanoScope III Controller Scanning mode in tapping mode (Veeco Instruments Inc., New York, NY, USA), before being immersed in the electrolyte (SCPS). The roughness of the HDGS substrate before and after applying one dip step of U(600), U(900), and U(2000), and one and three dip steps of U(600):3.45TEOS, U(900):3.45TEOS and U(2000):3.45TEOS were examined by AFM.

### 2.8. Characterization of Corrosion Performance of Hybrid Coatings on HDGS Samples in Contact with SCPS by Open Circuit Potential and Polarization Resistance Measurements

The corrosion behavior of the HDGS coated samples with the different hybrid materials was studied in solutions simulating the concrete interstitial electrolyte (simulated concrete pore solutions (SCPS)) and contaminated with 1 wt. % of chloride ions (SCPS + 1 wt. % Cl^−^). SCPS were prepared according to the literature [41,42] by adding analytical reagent grades 0.2 M KOH to a Ca(OH)_2_ saturated solution previously prepared with distilled water. A final solution with a pH = 13.2 was obtained and after 8 days, 1 wt. % of chloride ions was added in the form of sodium chloride. This medium was prepared in order to induce the corrosion of the substrate. According to Moreno et al. [42], the critical chloride concentration reported to induce corrosion of reinforcing steel in SCPS, with pH values of 12.5 and 13.9, was of 0.02 wt. % and 1 wt. %, respectively. Since the pH of the SCPS used in this work was above 12.5, a value of 1 wt. % was chosen to ensure that the chloride content was above the critical chloride concentration.

**Figure 3:**
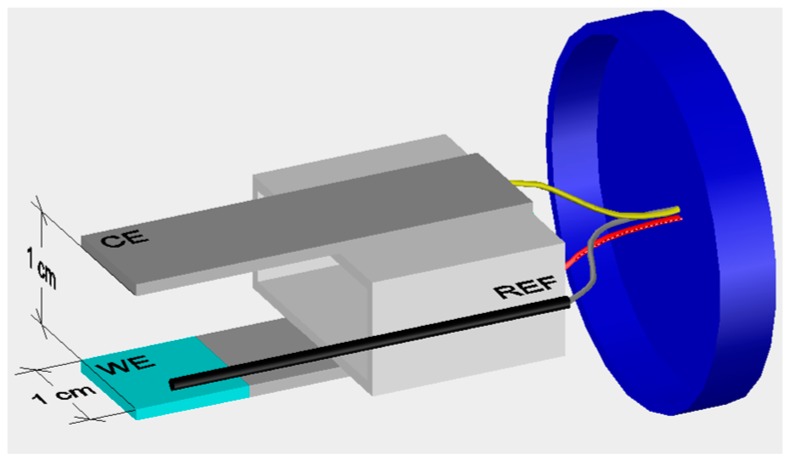
Electrochemical cell for polarization resistance measurements: WE, coated HDGS with the different hybrid materials; CE, stainless steel; REF, Ti/TiO_2_ (adapted from [43]).

The corrosion behavior of the HDGS coated with the different hybrid materials was assessed by open circuit potential (OCP) and polarization resistance (R_p_) measurements. All measurements were taken at room temperature. The measurements were performed with a three-electrode electrochemical cell (Figure 3) system using an established protocol [43,44,45]. The working electrode (WE) was a HDGS plate with an active area of 2 cm^2^ coated with the different hybrid materials. The counter electrode (CE) was a stainless steel (SS, type 316L) plate. The edges of both of the electrode plates, as well the non-active area and connecting zones, were protected with dual-component epoxy resin (Araldite^®^). The set of two electrodes was fixed in plastic lids that fit in a 100 mL polyethylene flask (Normax) [43]. A titanium wire (Ti/TiO_2_) with a length of about one centimeter was used as reference electrode (REF) [43,46]. The electrodes were connected to an isolated copper cable and the cutting zone of the tip of the titanium electrode was covered with epoxy resin (Araldite^®^). For comparison purposes, cells with non-coated HDGS WE electrodes were prepared and used as a reference, hereafter referred generically as control.

The R_p_ values were estimated by the potentiostatic method using a potentiostat/galvanostat (Voltalab PGZ 301, (Radiometer Analytical—Hach Company, Loveland, CO, USA)). A small anodic potential pulse (ΔE = +10 mV vs. reference electrode) was applied during 100 s, starting from open circuit potential values. The current vs. time transient was recorded and the ohmic drop was calculated and then subtracted from the measured R_p_ value [44,45,47].

## 3. Results and Discussion

### 3.1. NMR Analysis

**Figure 4:**
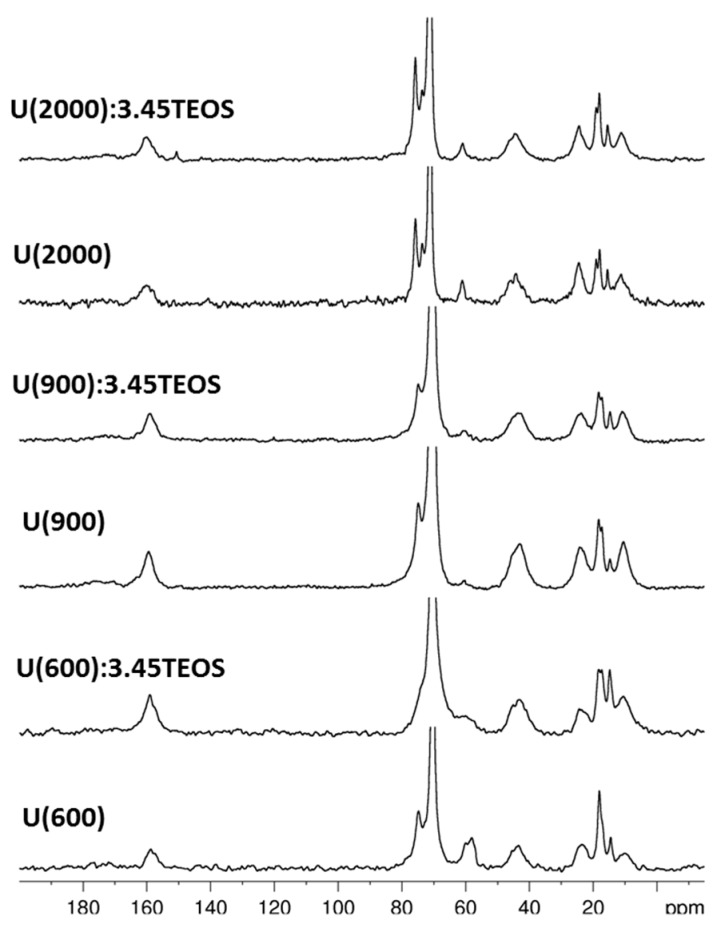
^13^C CPMAS spectra of hybrid samples with and without TEOS addition.

Structural features of the hybrid materials were investigated by multinuclear solid state NMR. The ^13^C CPMAS spectra of the hybrid samples with and without TEOS are shown in Figure 4 and the assignments of the signals are reported in Table 2.

**Figure 5:**
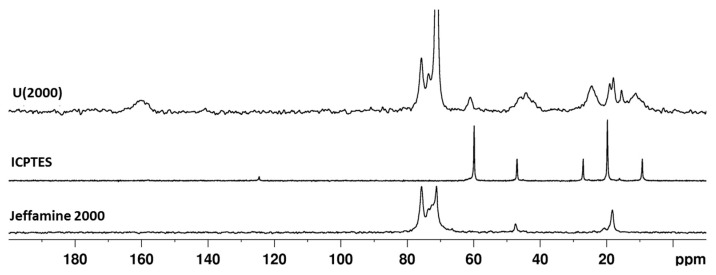
^13^C CPMAS spectra of hybrid sample U(2000) 1:4.16 and pure reagents (ICPTES and Jeffamine^®^ 2000).

**Figure 6:**
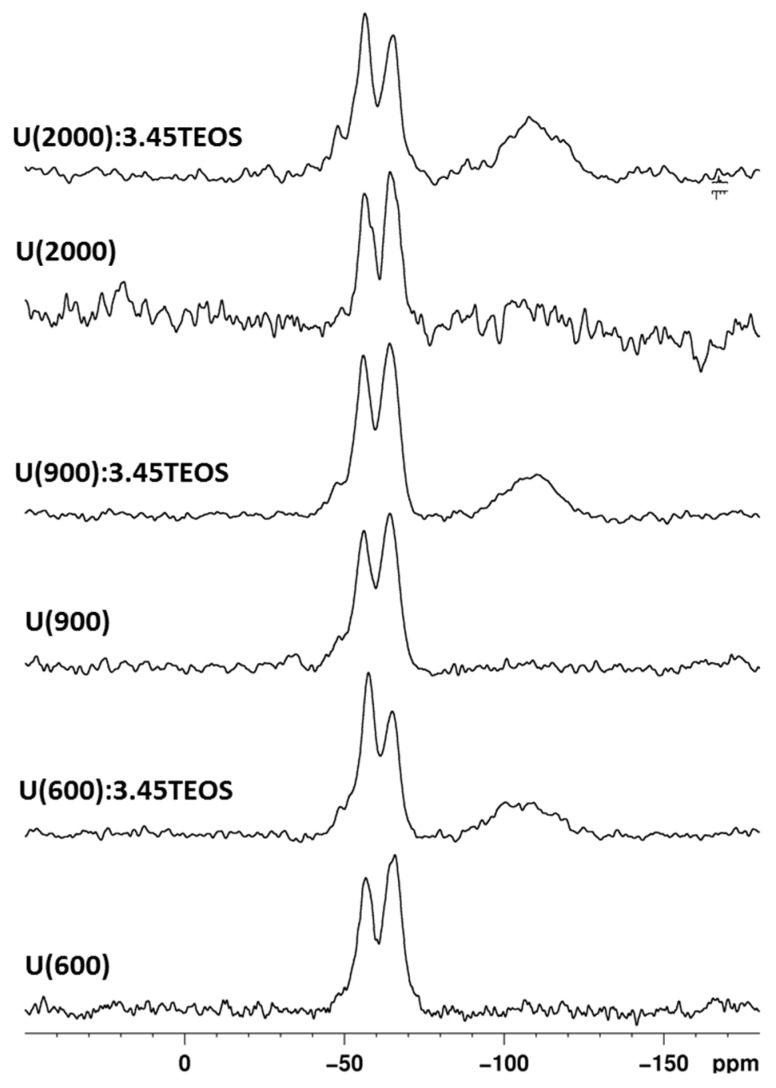
^29^Si MAS NMR spectra of hybrid samples prepared with and without TEOS.

The final materials give rise to carbon spectra characterized by sharp peaks due to the mobile PPG/PEG chains of the Jeffamine^®^ molecules and broad peaks produced by the short organic chains and the unreacted alkoxide groups of the silane. The spectra of samples with and without TEOS result superimposable, suggesting the hydrolysis of the ethoxide groups belonging to the tetra-alkoxysilane. Figure 5 reports the comparison among the spectra of Jeffamine^®^ 2000, ICPTES and the corresponding hybrid sample (U(2000)); since the pristine reagents are liquid, a small shift with respect to their signals in the solid state is expected. In the ICPTES spectrum, the peak attributed to the carbon atom in the isocyanate group (–N=C=O) is found at approximately 125 ppm. In the spectrum of the hybrid sample (U(2000) (Figure 6), the absence of the N=C=O ICPTES peak and the broad signal at about 160 ppm clearly shows that the reaction between the polymer amino groups and the N=C=O end group of ICPTES took place.

Nevertheless, the broad resonance at 160 ppm cannot be attributed to a single component but appears to be the result of several overlapped signals. According to the molar ratio among the reagents (Table 1), the amount of isocyanate groups is over-stoichiometric with respect to the Jeffamine terminal NH_2_ groups and, potentially, only half of the N=C=O groups can react with the amino groups of the Jeffamine molecule. Therefore, the residual isocyanate groups may react with ethanol leading to the formation of the urethane function. For assessing this hypothesis, ICPTES and Jeffamine^®^ 600 (selected according to the lower viscosity) have been mixed in the NMR tube in 1:4.16 molar ratios with deuterated ethanol and the ^13^C-NMR spectra have been acquired on the solution just after mixing the reagents, after 24 h aging and with the addition of citric acid (Figure S1, Supplementary Materials).

The ^13^C-NMR spectrum, recorded on the mixture immediately after mixing, shows a signal at 159 ppm and a less intense resonance at 157 ppm, respectively attributed to the –HNCONH– and –HNCOO-bridges [51,52] formed by reaction of the isocyanate groups of ICPTES. After 24 h, the decrease in intensity of the peak at 123 ppm due to N=C=O is clear, and the two resonances at 159 and 157 ppm present similar intensity. From these results, it can be concluded that both urea and urethane bridges are immediately created after the mixing of reagents. However, the formation of the –HNCONH– bridge is favored. It should be noted that the stoichiometric conditions used here caused the formation, during the aging process, of a valuable amount of urethane functions. The stability of the formed urea and urethane bridges has been evaluated by adding citric acid to the aged mixture. The carboxylic acid addition leads to the appearance in the spectrum of the carboxy groups signals at 170.4, 172.6 and 175.6 ppm, but does not affect the –HNCONH– and –HNCOO– signals, since the only variation is the increase of urethane groups by further isocyanate group consumption.

The quantitative analysis of the carbonyl band in the solid state ^13^C spectra of hybrid samples (Figure 4) is limited by the unsatisfactory signal-to-noise ratio (S/N). In order to better point out the signal components in the range 150–170 ppm (Figure 4), a ^13^C CPMAS spectrum was recorded on a selected sample with very high number of scans (10 k scans) and the profile fitting analysis was performed on the low-field part of the spectrum which presents more resolved signals and improved S/N (Figure S2, Supplementary Materials). The carbonyl band appears to be the result of four overlapping signals, two intense resonances at 159 ppm (47%) and 157 ppm (26%) and two signals at 154 (16%) and 162 ppm (11%). The clear assignment of the latter components is still under investigation but, in agreement with a previous FTIR study pointing out the presence of several components in the amide band of ureasilicate samples [53], that may be related to chains with different hydrogen bonding interactions.

A weak and broad resonance is also detected at about 172 ppm (Figure S2, Supplementary Materials), which can be attributed to the carboxylate functions of citric acid in agreement with the NMR study in solution (Figure S1, Supplementary Materials). The ^13^C spectra of hybrid samples (Figure 4 and Table 2) reveal the signals of residual ethoxyde groups suggesting that the organosilane hydrolysis is not complete in spite of the hydrolysis ratio used in the hybrid synthesis (Table 1). The content of citric acid in the mixtures is about 10% with respect to the whole silane amount (Table 1) and the complexation of the Si units by citric acid cannot be excluded, taking into account the well-known reactivity of carboxylic acids towards silicon alkoxides [54].

In the hybrid samples (Figure 4), the signals of PEG/PPG moieties appear unchanged when compared to the pristine Jeffamine^®^ molecules. The only exception is with Jeffamine^®^ 2000. The resonances at around 70–75 ppm change both in shape and intensity, when comparing the spectra of Jeffamine 2000 and U(2000) (Figure 5). Since these signals are not related to reactive parts of the molecules, the conformation rearrangement of the chains in the hybrid could be responsible for peak changes. Previous observations on the evolution of the FTIR signal of oxyethylene chains [53] pointed out that, in the hybrids, the chains partially lose the original helical conformation, acquiring a less ordered structure.

The ^29^Si NMR spectra were recorded on hybrid samples using both CP (Figure S3, Supplementary Materials) and SP (Figure 6) sequences; the Si units are labeled using the common NMR notation: Q^n^ and T^n^ are tetrafunctional SiO_4_ and trifunctional SiCO_3_ units, respectively and n is the number of siloxane bridges. The results of the profile fitting analysis of the ^29^Si MAS NMR spectra are reported in Table 3.

The ^29^Si NMR spectra (Figure 6) shows the typical signals of T units (−50 ÷ −70 ppm) due to ICPTES, and Q units (−90 ÷ −120 ppm) for samples prepared with TEOS addition. Hybrid samples prepared without TEOS addition show only T^2^ and T^3^ species. The degree of condensation (DOC) calculated according to the following equation [55]:

(1) $$\mathrm{DOC}=\;\frac{\left(T^{1}+2T^{2}+3T^{3}+2Q^{2}+3Q^{3}+4Q^{4}\right)}{3\left(T^{1}+T^{2}+T^{3}\right)+4\left(Q^{2}+Q^{3}+Q^{4}\right)}$$

is around 85% (Table 3). With TEOS addition, the T region is characterized by the signals of T^1^, T^2^, and T^3^ units, and the Q region presents the signals of Q^2^, Q^3^ and Q^4^ units; generally, the DOC values are very similar to those of the samples prepared without TEOS (Table 3). The ^29^Si NMR study confirms the reduced extent of hydrolysis-condensation of the sol-gel network as suggested by the ^13^C-NMR spectra.

The Q/T ratio reported in Table 3 assesses the amount of TEOS incorporated into the hybrid networks. The calculated values are different from the nominal ones; only the sample prepared with high MW of Jeffamine^®^ shows an amount of Q units closer to the expected value (Q/T 0.8). In order to prove that the Q units were not underestimated as a consequence of the MAS experiment conditions, these results were validated acquiring the MAS spectra with increasing the relaxation time parameter.

Besides the Q/T ratio obtained by the quantitative analysis, the Q units are not clearly visible in the ^29^Si CPMAS spectra (Figure S3, Supplementary Materials), contrariwise to CP spectra usually recorded with the same parameters on sol-gel hybrid organic/inorganic samples [55]. Work is in progress to clarify this result, which indicates poor magnetization transfer efficiency in the CP experiments, resulting from large distances among protons and TEOS-derived units in the network. One possible explanation could be the segregation of Q domains as a consequence of TEOS complexation with citric acid. The resulting silicate units would belong to the Q units region but contribute to creating the large quantity of Q^3^ and Q^2^ units, thus decreasing the overall DOC (Table 3).

### 3.2. Glow Discharge Optical Emission Spectroscopy (GD-OES)

GD-OES was used to obtain quantitative composition profiles in order to investigate the thickness of the hybrid coatings as a function of the number of layers deposited. The depth profiling chemical composition of every coating applied on HDGS was determined by GD-OES according to ISO 16962:2005(E). The detected elements were Zn, Fe, Si and C. The hybrid thickness was obtained from the difference between the depth found for the coated sample (zinc layer depth + the hybrid depth) and the uncoated sample (zinc layer depth). Table 4 shows the depth profile obtained for all samples studied.

GD-OES results show that Si was detected in a range of 21 to 30 μm of coating depth (Table 4). The thickness of the hybrid coatings was in a range between 2 to 11 μm. With exception of U(2000):3.45, Table 2 shows that, compared to the deposition of one layer, the coating thickness duplicates when three layers are deposited.

### 3.3. Electrochemical Impedance Spectroscopy (EIS) Analysis

**Figure 7:**
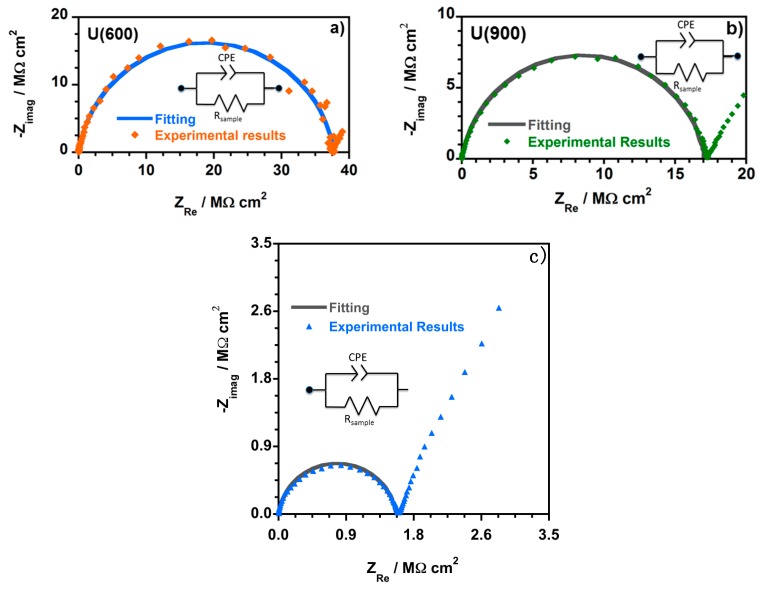
Typical complex plane impedance plots and the respective fitting obtained for hybrid films: (**a**) U(600); (**b**) U(900); and (**c**) U(2000) prepared with a molar ratio of Jeffamine:ICPTES = 1:4.16 (with the EEC as inset used to analyze the EIS response).

Figure 7 shows the Nyquist complex impedance plots obtained from the EIS analysis of the film samples, namely U(600), U(900) and U(2000) synthesized with a molar ratio of Jeffamine:ICPTES equal to 1:4.16. The electrical equivalent circuit (EEC) used to describe the observed impedance response of each hybrid is shown as an inset in each Nyquist plot.

**Figure 8:**
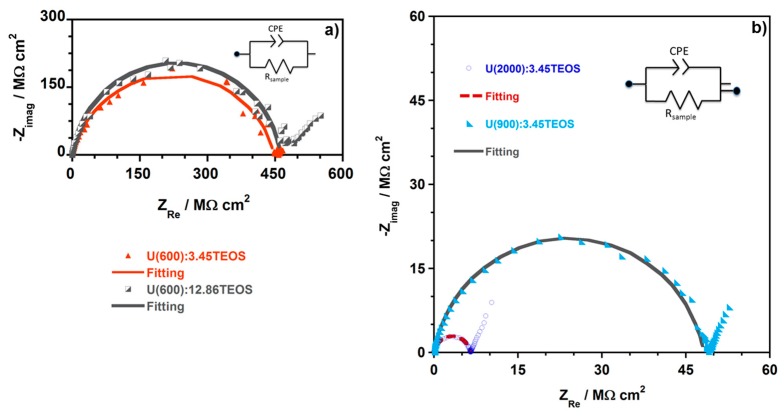
Typical complex plane impedance plots and the respective fitting obtained for hybrid films prepared with a molar ratio of Jeffamine:ICPTES = 1:4.16 and enriched with TEOS: (**a**) U(600):3.45TEOS and U(600):12.86TEOS; and (**b**) U(900):3.45TEOS and U(2000):3.45TEOS. The EECs used to analyze the EIS response are inset.

Figure 8 shows representative examples of the Nyquist plots obtained for U(X) samples enriched with different ratios of TEOS, namely U(600):3.45TEOS, U(900):3.45TEOS and U(2000):3.45TEOS.

Figure 7 and Figure 8 show that, at higher frequencies, a semicircle that intercepts the *x*-axis is present in all the Nyquist plots. The amplitude of the semi-circles changes with the sample composition. This is assigned to the electric properties, such as conductivity and capacitance, of the hybrid films. Data obtained at lower frequencies describe a line suggesting another electrochemical process, which is attributed to the Au | hybrid film interface [32]. The semicircles in the Nyquist plots (Figure 7 and Figure 8) show a depressed form, and so the analysis of all the impedance responses was based on EEC where constant phase elements (CPE) were used instead of pure capacitance. The impedance of a CPE is known by [56]:

Z_CPE_ = 1/[Q(jω)^α^] (2)

where α and Q represent parameters regardless of the frequency [57]. When α = 1, Q represents the capacity (F/cm^2^) while if α ≠ 1, Q has units of S^α^/Ωcm^2^ and the system shows a behavior that is linked to the surface heterogeneity [57,58,59].

A resistive–capacitive parallel circuit was considered and the impedance for the EEC is given by [57]:

Z_CPE_ = R_Sample_/[1 + (jω)^α^QR_Sample_] (3)

R_Sample_ represents the resistance in parallel with the CPE. The CPE parameter Q cannot be equated to the interfacial capacitance (C_eff_). Therefore, the C_eff_ was estimated using the relationship developed by Brug et al. [57,58]:

C_eff_ = [QR_sample_^(1−α)^]^1/α^ (4)

C_eff_ values were calculated according to Equation (4). Normalized resistance (R/Ω·cm^2^), normalized capacitance (C/nF·cm^−2^) and conductivity (σ/S·cm^−1^) were also determined. The R and C values were normalized to cell geometry dimensions. The obtained values were calculated using the following equations (where A_Au_ is the area of the gold electrodes and d_Sample_ the thickness of the analyzed OIH film sample).

R = R_Sample_ × A_Au disc_ (5)

C = C_eff_/A_Au disc_ (6)

σ = (d_Sample_/A_Au disc_)/R_Sample_ (7)

ε_r_ = (C_eff_ × d_Sample_)/ε_o_ × A_Au disc_ (8)

Only the electrochemical process at high frequencies was fitted and the EEC used to model the Nyquist plots for all the hybrid contain a simple CPE (Q/S^α^ Ω^−1^·cm^−2^) and a Resistance (R_sample_**/**Ω·cm^2^), which is associated to the hybrid film resistance. Five measurements were performed for each hybrid sample. However, only one representative example among the five measurements performed is shown (Table 5). The obtained fitting parameters, namely R_Sample_, Q (represented by CPE in the EEC) and α as well as the percentage of error associated to each element are presented in Table 5. The data obtained at lower applied frequencies describe a line suggesting another electrochemical process. This process is assigned to Au|hybrid film interfacial phenomena and a simple R-C EEC describes this part of the Nyquist plot. However, the fitting was not executed since it was not relevant for the understanding of the electrochemical behavior of hybrid films. The solid lines in all the Nyquist plots correspond to the fitted regions. The behavior observed at the different frequency ranges is a consequence of capacitance time constants with large differences that are linked to the charge transport across the hybrid and the charge relaxation that occurs at the interfaces.

C_eff_ values were calculated according to Equation (4) and as described in previous publications [32,36,60]. The resistance (R) and capacitance (C) values were normalized to cell geometry dimensions. The conductivity (σ/S·cm^−1^) was also determined.

**Figure 9:**
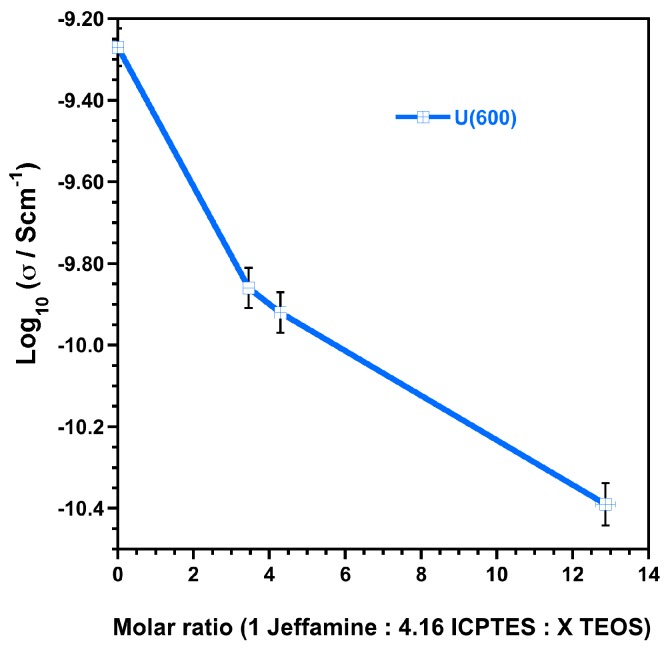
Average values of conductivity obtained at room temperature for U(600) film matrices prepared with different ratios of TEOS using a molar ratio of Jeffamine:ICPTES = 1:4.16.

Table 6, shows the average values of the logarithm of resistance (log R), conductivity (−log σ), C and ε_r_ obtained for all the samples of each hybrid film (uncertainty is expressed for 95% confidence). Figure 9 shows the average values of the conductivity (−log σ) (uncertainty is expressed for 95% confidence, (Table 6)) obtained for the hybrid film samples prepared with Jeffamine with a MW of approximately 600 g·mol^−1^ with different contents of TEOS (according to Table 1).

The conductivity values obtained for the U(X) samples prepared with a molar ratio of Jeffamine:ICPTES = 1:4.16 are lower when compared to the data obtained for samples prepared with a molar ratio of Jeffamine:ICPTES = 1:2 [32]. When TEOS is added, Table 6 and Figure 9 show that, as the content of TEOS increases, the conductivity keeps decreasing, the normalized R values increase and the ε**_r_** decreases. Moreover, excepting for the hybrid films based on U(2000) matrices, the normalized R values obtained are all above 10^7^ Ω·cm^2^ (Table 5), suggesting that these materials meet the necessary requirements to provide an effective corrosion protection [61,62].

The results show that the addition of TEOS to the hybrid matrices, which increases the inorganic component of the matrix and decreases the sol viscosity, improves the barrier properties of the ureasilicate matrices by increasing their resistance (and decreasing the conductivity); this evidence is in agreement with previous studies [31,37].

The capacitance is another parameter used for the characterization of the barrier protective properties. The values obtained for samples based on U(600) matrices (Table 5) generally increase when the content of TEOS increases. However, for higher MWs of Jeffamine^®^ this tendency was not found. Samples based on U(900) and U(2000) matrices showed lower capacitances when the content of TEOS increased. EIS data evidence that the molecular size of organic (Jeffamine^®^) component in the ureasilicate network has a major relevance on the dielectric properties. This emphasizes that the charge transport and relaxation processes occur predominantly across in the organic environment.

### 3.4. Atomic Force Microscopy (AFM)

**Figure 10:**
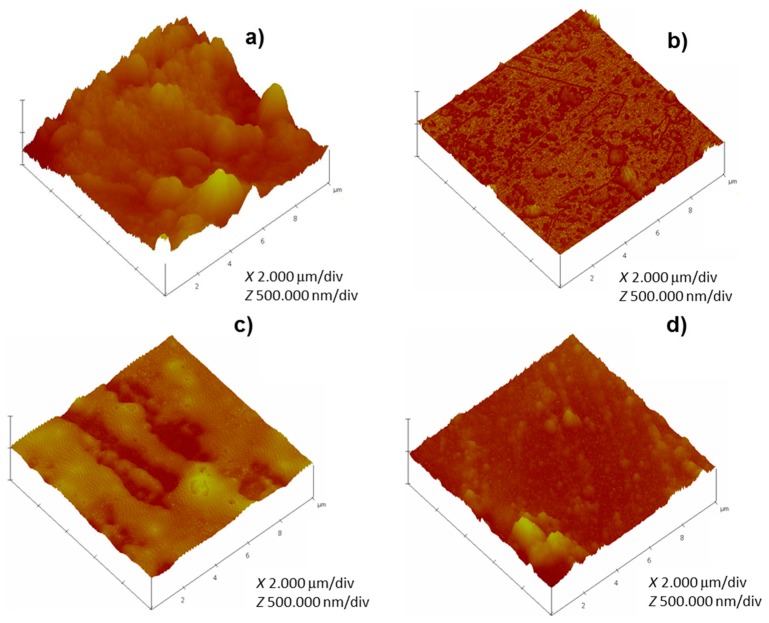
Atomic Force microscopy (AFM) topographic images (3D view) for: (**a**) control (uncoated HDGS); and HDGS samples coated with one layer of: (**b**) U(600):3.45TEOS; (**c**) U(900):3.45TEOS; and (**d**) U(2000):3.45TEOS.

The AFM analysis of the surface of HDGS coated samples were scanned on an area of 10 × 10 μm^2^. Figure 10 shows representative topographic images of samples coated with one layer of hybrid enriched with TEOS before immersion in SCPS.

As expected, coated samples displayed smoother surfaces compared to control (uncoated samples). Nevertheless, the coated surfaces were not uniform and the presence of defects was also detected (Figure 10). The coating method deposition used, as well as the curing process, may explain the presence of these agglomerates and defects [27,33,34]. The amplitude parameters used to characterize the topography of a surface are the average roughness (R_a_) and the root mean square roughness (R_q_). Table 6 shows the values of R_q_, R_a_, and R_max_ (maximum vertical distance between the highest and lowest data points within a given area) for the control and HDGS-coated samples. As expected, control shows higher R_q_ values (above 100 nm) and coated samples show lower R_q_ values, below or equal to 46 nm for samples without TEOS and below or equal to 35 nm for samples enriched with TEOS.

Generally, Table 7 shows that, as the MW of Jeffamine^®^ increases, the coating roughness (R_q_) increases. The introduction of TEOS during the synthesis leads to coatings with lower R_q_ values. This effect is due, probably, to the decrease of the viscosity of the sol hybrid sols by TEOS addition, which allows improved distribution on the metallic substrate.

U(900) samples are an exception, as the samples prepared with TEOS showed higher R_q_, R_a_ and R_q_ than samples prepared without TEOS. It is worth of noting that R_max_ obtained for samples prepared with Jeffamine^®^ of MW 2000 is very high, when compared to other samples (generally ≈ 3 times higher). Again this result can be related to the viscosity of the hybrid sols. Indeed, as the MW of Jeffamine^®^ increases the viscosity of the sols increase. Accordingly, for samples prepared with Jeffamine^®^ of MW ≈ 2000 g·mol^−1^, the high viscosity results in inefficient/poor sol distribution and leads to the formation of large agglomerates (defects). These defects present higher dimensions than the ones formed when MWs of Jeffamine^®^ are used.

The coating quality and surface roughness play a role on the corrosion behavior of metallic materials. An increase in the surface roughness increases the pitting susceptibility and corrosion rate which is according to the literature [63]. The AFM and the EIS results are in agreement and show that, generally, as the MW of Jeffamine^®^ increases, the roughness increases and the resistance of the hybrid coatings decreases which is also according to previous studies [27]. This tendency remains, even when the samples are enriched with TEOS. The results indicate that the corrosion behavior is not only dependent on coating quality but also on roughness, and the results have shown that samples with poor corrosion protection have high roughness, which is consistent with data found in the literature [27,63,64].

### 3.5. Open Circuit Potential (OCP) and Polarization Resistance (R_p_) Measurements

**Figure 11:**
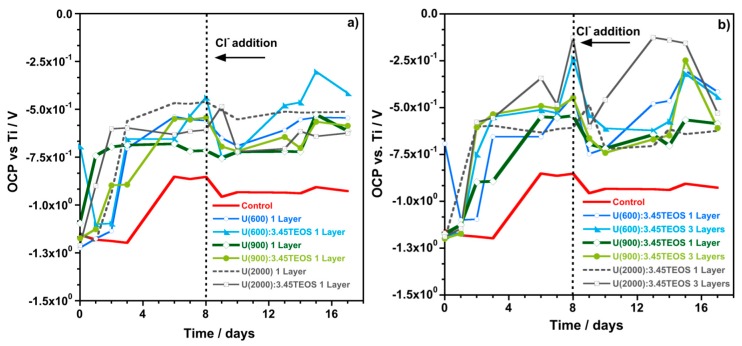
OCP variation over time recorded for the different coated HDGS sample cells kept embedded in SCPS for 17 days. Displayed graphs match to different urea-silicate matrices with and without TEOS: (**a**) U(X) and U(X):3.45TEOS samples deposited by one dip step (one layer); and (**b**) U(X):3.45TEOS deposited by three dip steps (three layers).

The OCP and R_p_ measurements were performed daily. The information extracted from OCP results indicates either a low or high probability of corrosion occurrence but it does not provide information on the corrosion rate [65,66,67]. The R_p_ measurements represent the instantaneous corrosion current density. These measurements allow to assess the condition of the embedded steel reinforcement related to its corrosion [65]. Figure 11 shows the OCP values obtained for each sample. Generally, coated samples show an increase in the OCP variation over time and the values are always higher than those obtained for the control sample. This behavior indicates that coated samples have low probability of corrosion occurrence (higher OCP values) compared to control samples (lower OCP values). After chloride addition the initial drop in the OCP values can be observed in all cases followed by an increase over time for coated samples, whereas the control sample display almost constant OCP values. Again, the OCP results indicate that coated samples, even in the presence of chloride ions, have lower probability of corrosion occurrence compared to control samples.

Table 8 shows the R_p_ values obtained on the Days 1, 7, 9 and 16 of immersion. Generally, coated samples show an increase in the R_p_ values after seven days of immersion (Table 8), whereas the control samples show a significant decrease. Furthermore, low R_p_ values were obtained for the control samples on Day 7 when compared to all the coated samples that showed higher R_p_ values (between 10^3^ and 10^5^ Ω·cm^2^). The behavior displayed by the control samples is explained by the zinc corrosion process in alkaline environments [16,66,67]. For high pH values, the passivation of the surface of the substrate is difficult to reach and Zn dissolution continues until all the zinc has been dissolved. After Cl^−^ addition a sharp decrease of R_p_ values was registered for control samples and samples coated with three layers. Nevertheless, the values remained above those obtained for control samples. Improved results (high R_p_ values) were obtained by coating HDGS with only one layer of U(900) with TEOS and U(600) with and without TEOS, even after Cl^−^ addition.

After sixteen days of immersion, Table 8 shows that the R_p_ values of the control samples did not change significantly and are considerably lower than those measured for coated samples. The results indicate that, on Day 16, the instantaneous corrosion current density reported for control samples is much higher compared to the values obtained for samples coated with one or three layers of hybrid with or without TEOS. Samples coated with one layer of U(2000):3.45TEOS and U(600):3.45TEOS provided higher and poorer corrosion protection, respectively. Samples coated with three layers of U(600):3.45TEOS and U(900)3.45TEOS provided the highest and the poorest corrosion protection, respectively.

Generally, AFM and R_p_ data show that, by TEOS incorporation into the hybrid matrices, the samples show improved corrosion behavior and lower roughness values (R_a_ and R_q_). This study also points out that the OCP, R_p_ and EIS results are generally in agreement and samples enriched with TEOS, regardless the MW of Jeffamine^®^ used, show improved results in terms of higher resistance values and improved corrosion behavior. A higher degree of crosslinking was expected for samples enriched with TEOS, which would explain the improvement of the corrosion behavior. On the contrary, the NMR study pointed out that the TEOS addition does not lead to relevant structural changes in the hybrid network. Therefore, the results suggest that the tetraalkoxysilane (TEOS) could have a role as a coupling agent, providing improved affinity between the organic component and the metallic substrate by means of the interaction between Si-OH and metal-OH (native oxide layer of HDGS) [31]. Nevertheless, no further conclusions can be drawn.

## 4. Conclusions

The present work reports the effect of TEOS addition into ureasilicate matrices on the structural properties of the hybrid sol-gel materials and on the corrosion behavior in chloride-contaminated SCPS.

The NMR results point to conclusion that: (i) the reaction between the polymer amino groups and the N=C=O end group of ICPTES takes place; (ii) when using ICPTES in excess, urea and urethane bridges are created immediately after the mixing of the reagents, but the formation of the –HNCONH– bridge is favored; (iii) these bridges remain stable upon carboxylic acid addition; and (iv) the addition of TEOS seems not to affect the organic structure of the hybrid materials and does not increase the DOC of the hybrid materials. The NMR study also suggest the presence of several components in the amide band of ureasilicate samples due to polyethereamine chains with different hydrogen bonding interactions. Moreover, the possible segregation of TEOS-derived domains can be assumed on the basis of cross polarization experiments. Additional studies should be conducted in order to understand these evidences.

EIS data show that, with increasing TEOS content, the resistance of the hybrid films increases. The electrochemical results obtained from monitoring cells (OCP and R_p_ data) on HDGS coated samples exposed to SCPS (before and after chloride ion addition) show that all the samples display improved performance when compared to the control (the uncoated HDGS sample). OCP, R_p_ and EIS results are, generally, in agreement, and the samples enriched with TEOS, regardless the MW of Jeffamine used, show superior corrosion behavior when compared to the hybrid samples without TEOS. Taking into account the structural study, it can also be concluded that the corrosion behavior is dependent on the coating quality, in particular the surface roughness. Indeed, the hybrid samples with superior corrosion protection display low roughness parameters and roughness decreases with TEOS addition. Moreover, the metal-coating interaction could be improved by increasing the inorganic moiety in U(X) hybrids. The results point to the conclusion that the ureasilicate coatings enriched with TEOS have favorable properties to be employed as pre-treatments to reduce corrosion activity during the initial stages of contact of the HDGS samples with highly alkaline environments (pH > 12.5) and in the presence of aggressive species such as chloride ions.

Further work has to be performed in the future in order to understand medium- and long-term performance of these hybrid systems. Moreover, studying in depth the metal-coating interface appears a tool for a full understanding of the hybrid coatings behavior. The auspicious progress demonstrated by this study suggests that these materials can be successfully used in the field of functional coatings. Studies on different substrates and environments should be carried out because these materials may potentially be used in a wide number of areas, such as automotive field, optical and photovoltaic devices, and consumer goods.

## Figures and Tables

**Table 1:** Molar ratios of the different hybrid films and coatings synthesized.

Samples	MW of Jeffamine	Molar Ratios			
		Jeffamine:ICPTES	Jeffamine:TEOS	H_2_O:Jeffamine	Jeffamine:Citric Acid
U(600)	600	1:4.16	n.a.	29.67:1	1:0.22
U(600):3.45TEOS			1:3.45		
U(600):4.29TEOS			1:4.29		
U(600):12.86TEOS			1:12.86		
U(900)	900	1:4.16	n.a.	29.67:1	1:0.22
U(900):3.45TEOS			1:3.45		
U(2000)	2000	1:4.16	n.a.	29.67:1	1:0.22
U(2000):3.45TEOS			1:3.45		

**Table 2:** ^13^C-NMR chemical shifts (δ (ppm)) and assignments based on literature [48,49,50,51].

δ (ppm)	Assignment
11.6	Si-CH_2_ (ICPTES)
15.7	CH_3_·PPG (Jeffamine^®^)
18.0	CH_3_·terminal PPG (Jeffamine^®^)
20.0	CH_3_·OEt (ICPTES)
24.7	CH_2_, propyl chain (Jeffamine^®^)
44–46	N-CH_2_/NCH (ICPTES)
61.0	CH_2_·OEt (ICPTES)
70.4	OCH_2_·PEG (Jeffamine^®^)
72–76	OCH·OCH_2_·PPG (Jeffamine^®^)
124.8	N=C=O (ICPTES)
160.2	C=O (HN-CO-NH/NH-COO-bridge, hybrid)

**Table 3:** ^29^Si NMR chemical shifts, assignments and relative amounts of silicon species (values are reported with a 95% confidence level).

Si Unit	T^1^	T^2^	T^3^	Q^2^	Q^3^	Q^4^	DOC	%T	Q/T
δ (ppm)	−49.5	−56.9	−64.4	−89.5	−99.3	−110.0	-	-	-
U(600)	7.6	36.4	56.0	-	-	-	82.8	100.0	-
U(600):3.45TEOS	5.7	36.7	32.0	-	15.9	9.6	80.3	74.5	0.3
U(900)	9.6	38.8	51.6	-	-	-	80.7	100.0	-
U(900):3.45TEOS	6.9	29.4	39.5	-	5.2	18.9	85.0	75.9	0.4
U(2000)		42.1	57.9	-	-	-	86.0	100.0	-
U(2000):3.45TEOS	3.6	27.7	32.9	7.2	12.5	16.0	81.6	64.3	0.6

**Table 4:** Depth of the hybrid coating and zinc layer and thicknesses obtained for each sample.

Samples	Number of Layers	Depth/μm (Zn Layer)	Depth ^a^/μm (Hybrid + Zn Layer)	Hybrid Thickness/μm (Hybrid + Zn Layer − Zn Layer)
Control	-	19	-	-
U(600):3.45TEOS	1	-	21	2
	3		25	4
U(900):3.45TEOS	1	-	23	4
	3		27	8
U(2000):3.45TEOS	1	-	28	9
	3		30	11

^a^ Hybrid thickness obtained according to ISO 16962:2005(E).

**Table 5:** EIS data fitting of the U(X) hybrid film samples with different ratios of TEOS.

Hybrid Sample	R_Sample_/Ω·cm^2^	CPE (Q)/S^α^·Ω^−1^·cm^−2^	α	Χ^2^
U(600)				
1:4.16:0 ^(a)^	3.78 × 10^7^ (±0.85%)	1.42 × 10^−11^ (±3.14%)	0.90 (±0.29%)	7.09 × 10^−3^
1:4.16:3.45 ^(a)^	4.48 × 10^8^ (±3.71%)	1.59 × 10^−11^ (±3.71%)	0.85 (±0.47%)	7.47 × 10^−3^
1:4.16:4.29	3.15 × 10^8^ (±1.30%)	2.75 × 10^−11^ (±3.06%)	0.90 (±0.32%)	2.30 × 10^−2^
1:4.16:12.86 ^(a)^	9.42 × 10^5^ (±0.19%)	3.92 × 10^−10^ (±3.23%)	0.76 (±0.40%)	1.64 × 10^−2^
U(900)				
1:4.16:0 ^(a)^	1.71 × 10^7^ (±0.72%)	4.36 × 10^−11^ (±3.14%)	0.90 (±0.31%)	1.05 × 10^−2^
1:4.16:3.45 ^(a)^	4.82 × 10^7^ (±1.14%)	1.61 × 10^−11^ (±3.91%)	0.89 (±0.41%)	9.95 × 10^−3^
U(2000)				
1:4.16:0 ^(a)^	1.52 × 10^6^ (±0.59%)	5.98 × 10^−11^ (±4.30%)	0.91 (±0.42%)	7.23 × 10^−3^
1:4.16:3.45 ^(a)^	6.50 × 10^6^ (±0.44%)	2.32 × 10^−11^ (±2.68%)	0.93 (±0.24%)	3.92 × 10^−3^

Notes: ^(a)^ EIS data fitting at high frequencies using the EEC, as shown as inset in correspondent Nyquist plots, Figure 7 and Figure 8.

**Table 6:** Electrical properties (average values) obtained for the U(X) hybrid film samples with different ratios of TEOS.

Hybrid Sample	log (R/Ω·cm^2^)	−log (σ/Scm^−1^)	C/pF·cm^−2^	ε_r_
U(600)				
1:4.16:0	7.47 ± 0.01	8.31 ± 0.01	8.12 ± 0.05	13.4 ± 0.1
1:4.16:3.45	8.57 ± 0.03	9.86 ± 0.03	8.69 ± 0.42	15.8 ± 0.8
1:4.16:4.29	8.41 ± 0.06	9.92 ± 0.06	20.5 ± 2.0	9.40 ± 1.10
1:4.16:12.86	8.56 ± 0.04	10.5 ± 0.1	30.8 ± 0.6	3.70 ± 0.10
U(900)				
1:4.16:0	7.16 ± 0.06	8.20 ± 0.03	25.1 ± 0.1	23.9 ± 0.1
1:4.16:3.45	7.57 ± 0.02	8.50 ± 0.02	8.80 ± 0.07	11.6 ± 0.4
U(2000)				
1:4.16:0	6.13 ± 0.09	7.20 ± 0.16	31.0 ± 3.5	29.7 ± 1.9
1:4.16:3.45	6.67 ± 0.11	7.65 ± 0.04	14.1 ± 0.2	15.3 ± 0.2

**Table 7:** Roughness parameters obtained for control and the different coated HDGS samples coated with one layer.

Sample	R_q_/nm	R_a_/nm	R_max_/nm
Uncoated HDGS	107	82	611
HDGS_U(600) 1 layer	14	11	106
HDGS_U(600):3.45TEOS 1 layer	12	7	164
HDGS_U(900) 1 layer	17	15	103
HDGS_U(900):3.45TEOS 1 layer	28	22	184
HDGS_U(2000) 1 layer	46	34	348
HDGS_U(2000):3.45TEOS 1 layer	35	25	326

**Table 8:** R_p_ values obtained for the different coatings prepared with different ratios of TEOS.

Samples	Number of Layers	Day 1	Before Cl^−^ Addition (Day 7)	After Cl^−^ Addition (Day 9)	Day 16
		(Ω·cm^2^) × 10^3^	(Ω·cm^2^) × 10^3^	(Ω·cm^2^) × 10^3^	(Ω·cm^2^) × 10^3^
Control		0.649 ± 0.188	0.023 ± 0.007	0.008 ± 0.002	0.009 ± 0.003
U(600)					
1:4.16:0	1	0.238 ± 0.042	21.5 ± 3.8	32.1 ± 5.6	153 ± 27
1:4.16:3.45	1	16.4 ± 4.8	14.6 ± 4.2	14.6 ± 5.3	0.024 ± 0.007
1:4.16:3.45	3	2.94 ± 0.53	32.6 ± 5.9	0.018 ±0.003	73.2 ± 13.2
U(900)					
1:4.16:0	1	3.67 ± 0.64	314 ± 55	0.040 ± 0.007	0.044 ± 0.008
1:4.16:3.45	1	0.004 ± 0.001	141 ± 21	354 ± 53.1	118 ± 18
1:4.16:3.45	3	0.192 ± 0.052	37.3 ± 10.1	0.035 ± 0.009	0.129 ± 0.035
U(2000)					
1:4.16:0	1	31.8 ± 0.5	12.1 ± 1.82	0.389 ± 0.058	0.424 ± 0.064
1:4.16:3.45	1	0.120 ± 0.033	5.78 ± 1.58	13.7 ± 3.8	257 ± 71
1:4.16:3.45	3	0.489 ± 0.134	145 ± 40	0.018 ± 0.005	0.557 ± 0.153

## Supplementary Materials

The following are available online at www.mdpi.com/1996-1944/10/3/306/s1. Figure S1: ^13^C-NMR of the mixture Jeffamine 600/ICPTES (1:4) in ethanol-*d*_6_ immediately after mixing and after 24 h and with subsequent addition of citric acid, Figure S2: Lowfield part of the ^13^C CPMAS spectrum of U(900):3.45TEOS, Figure S3: ^29^Si CPMAS NMR spectra of hybrid samples prepared with and without TEOS.
