# Supplementary material for: Hybrid Coatings Enriched with Tetraethoxysilane for Corrosion Mitigation of Hot-Dip Galvanized Steel in Chloride Contaminated Simulated Concrete Pore Solutions
Source: Materials (Basel). 2017 Mar 17;10(3):306. doi: 10.3390/ma10030306 (PMC5503348; doi:10.3390/ma10030306)
Supplement: Supplementary file 1 [file materials-10-00306-s001.pdf]

Supplementary Material

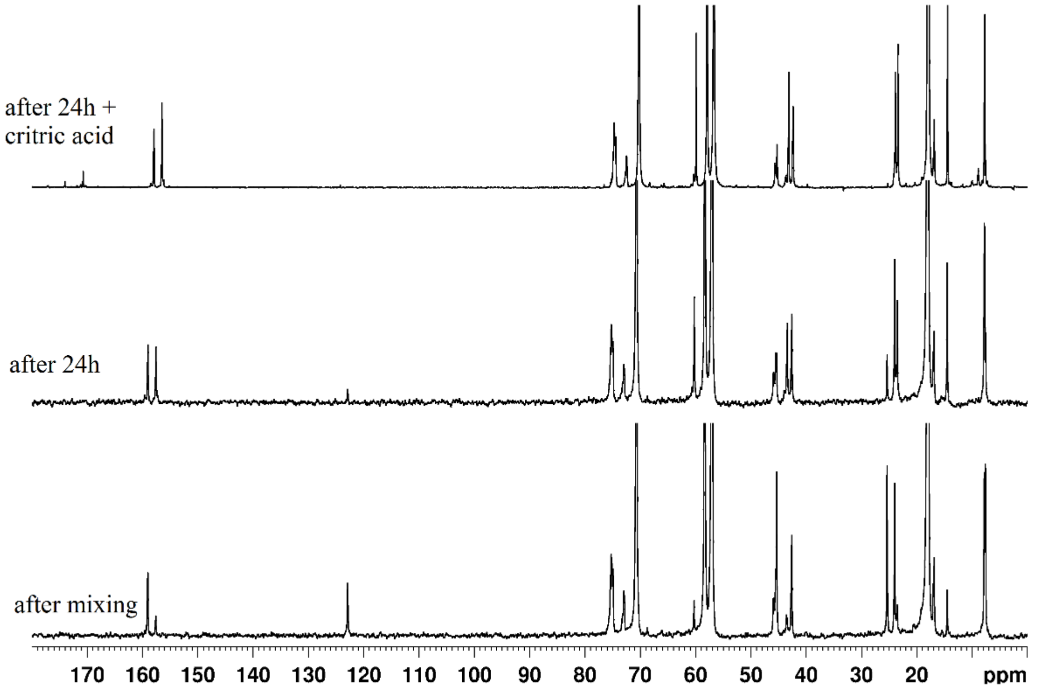

Figure S1. ^{13}C NMR of the mixture Jeffamine 600/ICPTES (1:4.16) in ethanol-d6 immediately after mixing and after 24h and with subsequent addition of citric acid.

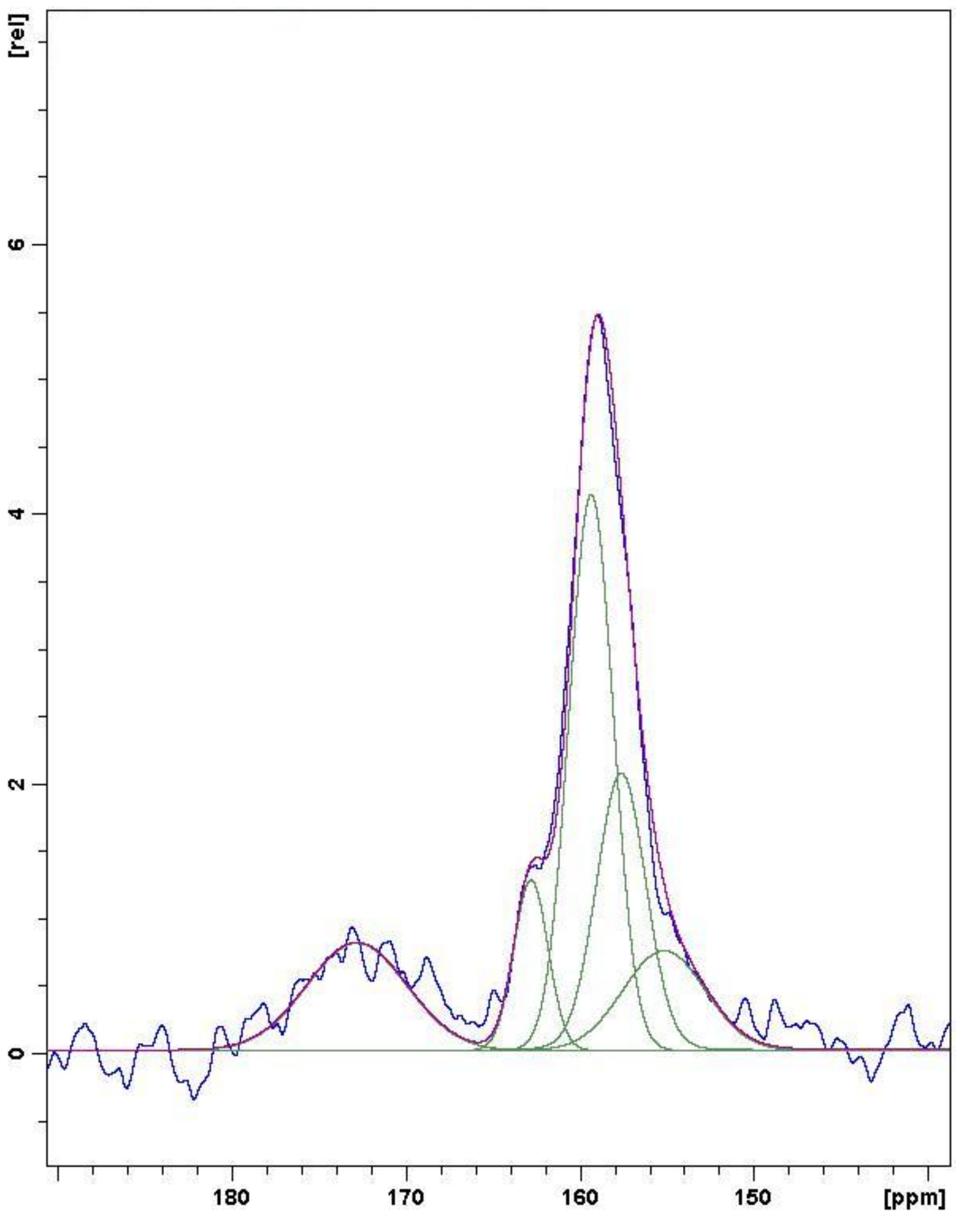

Figure S2. Lowfield part of the ^{13}C CPMAS spectrum of U(900): 3.45TEOS.

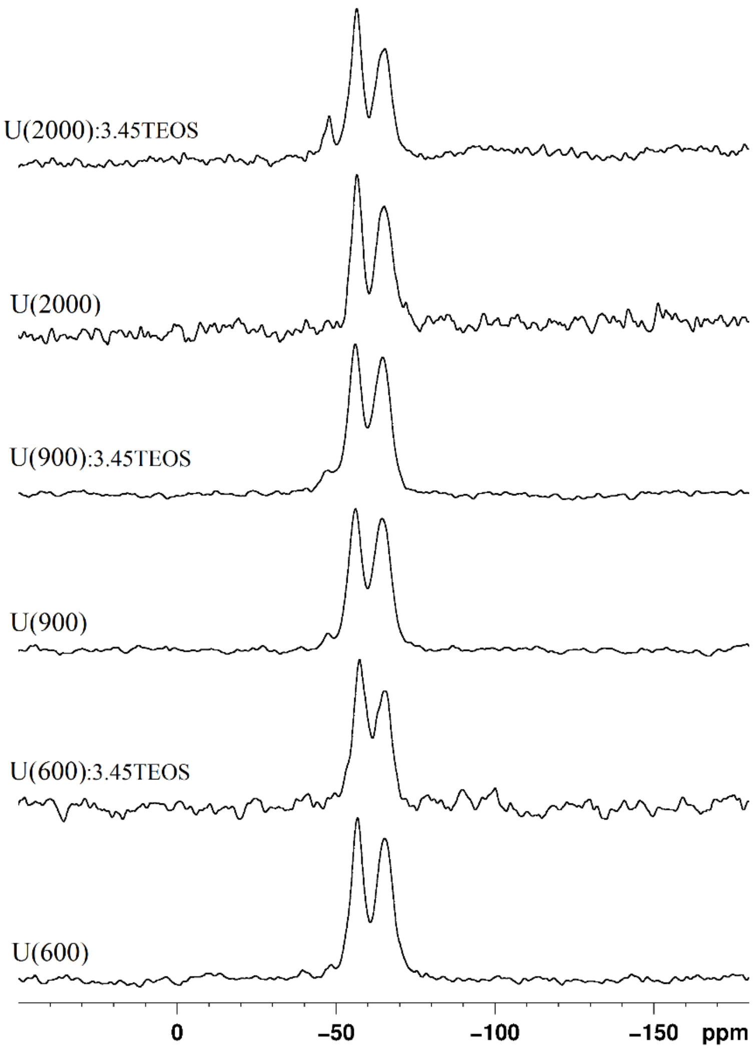

Figure S3. ^{29}Si CPMAS NMR spectra of OIH samples prepared with and without TEOS.
